# Translation and validation of the Breastfeeding Motivation Scale in China

**DOI:** 10.1186/s13006-023-00610-z

**Published:** 2024-01-04

**Authors:** Yanfei Yu, Lu Zhang, Ying Liu, Lan Zhang, Nafei Guo

**Affiliations:** Nursing Department, Shanghai First Maternity and Infant Hospital, Tongji University School of Medicine, No.2699, West Gaoke Road, Pudong New Area, Shanghai, 201204 China

**Keywords:** Breastfeeding, Motivation, Mother, Maternal, Reliability, Validity, Cross-cultural adaptation

## Abstract

**Background:**

There are several versions of the Breastfeeding Motivation Scale (BMS), which have been shown to measure maternal breastfeeding motivation, but there is not a Chinese version yet. The study aimed to translate the BMS into Chinese and subsequently assess its psychometric properties among Chinese mothers during the postpartum period.

**Methods:**

The study was composed of two phases. The translation of BMS closely followed the principals of good practices. Phase 1 included a comprehensive translation, back-translation, cross-cultural adaptation, and pretest to develop the Chinese version of the BMS. From 1 December 2021 to 1 July 2022, the Chinese version of the BMS was administered to 206 postnatal mothers in our maternity wards to assess its psychometric properties. Phase 2 involved psychometric property testing, including testing of the internal consistency, test–retest reliability, content validity, construct validity, convergent validity and discriminant validity.

**Results:**

Minor modifications in four items were recommended after translations. The Cronbach's α coefficient of the Chinese version of the BMS was .887, and the intraclass correlation coefficient was .897 (*P* < 0.001). The model fit was acceptable (χ2/*df* = 2.40, *P* < 0.001, RMSEA = 0.08, CFI = 0.91, IFI = 0.92 and TLI = 0.90) according to the confirmatory factor analysis. The composite reliability values corresponding to each latent variable were 0.733 ~ 0.926, and the average variance extracted values were 0.476 ~ 0.653. The correlations among the five measured variables were all lower than .85 and the square roots of average variance extracted from the variable were greater than the interconstruct correlations among the five measured variables in the model.

**Conclusions:**

The Chinese version of the BMS has good reliability and validity and provides a reliable assessment tool for measuring maternal breastfeeding motivation. It also provides support to develop culturally sensitive interventions for Chinese mothers’ who are breastfeeding.

**Supplementary Information:**

The online version contains supplementary material available at 10.1186/s13006-023-00610-z.

## Background

Most mothers are familiar with the benefits of breastfeeding. While Chinese mothers are provided with breastfeeding education by most healthcare providers during their postpartum hospital stay [1, 2], the exclusive breastfeeding rates under six months in China are quite low [3, 4] compared to the WHO recommendations [4–6]. Many Chinese mothers choose mixed feeding or exclusive formula feeding for their babies in the early postnatal period, or have difficulty sustaining exclusive breastfeeding [7]. Breastfeeding, like other health behaviors, is influenced by a complex mix of sociodemographic, biomedical, cultural, economic, geographical, and psychosocial factors that may prevent a mother from achieving these recommendations or her own breastfeeding goals [4, 8, 9].

Psychological factors are important predictors of breastfeeding initiation and continuation. For instance, a woman's sense of autonomy and self-efficacy have been found to predict more positive breastfeeding outcomes [10]. Similarly, a higher level of breastfeeding self-efficacy predicts continued breastfeeding at six months [11], and women’s breastfeeding empowerment positively affects the initiation and duration of breastfeeding [12]. The perception of infant attachment may help mothers adjust to parental role demands, which in turn may make mothers prefer breastfeeding [13].

Self-determination theory (SDT), developed by Ryan and Deci [14], is one of the leading contemporary motivational frameworks. It has been used to understand human motivation and behavior and posits an individual’s ability to satisfy their basic psychological needs with autonomy (i.e., the perception that one’s behavior is fully volitional), competence (i.e., the ability to feel effective, to influence the outcome), and relatedness (i.e., the feeling of attachment, importance and belonging with others), and influences their motivation and, in turn, their behavior [15]. SDT has been applied in several health behavior domains, such as physical activity [16], oral health [17], weight control [18], tobacco cessation [19] and breastfeeding [15, 20–22].

Is breastfeeding always the “right path”? Some mothers do not think so, which may be why breastfeeding rates fall far below national health objectives [20]. SDT may give us one opportunity to explore the underlying causes of this situation. Miri Kestler-Peleg et al. [20] developed the Breastfeeding Motivation Scale (BMS) based on SDT to explore the motivation for breastfeeding. The scale was developed in 2015 and was used among mothers in Israel, and it has been validated and shown to have sound reliability and validity. It is a promising instrument with which to measure maternal breastfeeding motivation and highlight the role of these breastfeeding motivations among mothers. The BMS was translated into a Turkish language version, and the Turkish validity and reliability of the scale are acceptable. The BMS has been a reliable tool to measure motivation to breastfeed in both primigravida and multigravida women in Israel [20] and Turkey [23, 24].

China is the world's largest developing country and has the largest population in the world. Approximately 10 million babies are born in China each year [25]. Based on the benefits of breastfeeding, it is important to know the motivation for breastfeeding and to improve breastfeeding initiation, exclusivity, and duration. Chinese is the world's most spoken language. However, no study validating the BMS in Chinese exists at present. Accordingly, the present study aimed to translate the scale into Chinese, and to subsequently assess the psychometric properties of the Chinese version of the BMS among Chinese mothers during the postpartum period.

## Methods

### Measures

#### Mothers’ sociodemographic and clinical data questionnaire

Mothers’ sociodemographic and clinical data were collected by investigators using the questionnaire developed by the researchers. The mothers’ sociodemographic and clinical data included (1) sociodemographic data, (2) gestational age, (3) pregnancy and birth history, (4) mode of birth, (5) medical history, and (6) neonatal situation.

#### BMS

The BMS was developed by Israeli academic Miri Kestler-Peleg and his team members [20] in 2015. The original English-language BMS initially contained 24 items, but after principal axis factor analysis, the developers removed item 1, "Breastfeeding is more convenient because there is no need to wash and sterilize bottles, and breastfeeding can be done anywhere, anytime". Therefore, the final BMS in English consists of 23 items covering 5 subscales: (1) enjoyment and bonding, (2) maternal self-perception, (3) significant others' pressure, (4) baby's health, and (5) instrumental needs. Each item is scored on a 4-point Likert scale ranging from 1 meaning “strongly disagree” to 4 meaning “strongly agree”. Respondents are asked to rate extent to which each item matched the reason they chose to breastfeed. The scale has no total score. As the subscale score increases, the motivation that represents that subscale also increases [23]. The Cronbach's α for each subscale of the BMS in English ranged from 0.62 ~ 0.93. The Cohen’s kappa consistency coefficients of the scale ranged from 87.5% to 100 percent.

### Participants, site and design

We conducted a cross-sectional study in Shanghai First Maternity and Infant Hospital, which is a high-level national maternity hospital in China. The hospital, affectionately known as the "Great Cradle of Shanghai" by Shanghai residents, handles approximately 30,000 deliveries a year. The instruments were applied during the mother’s hospital stay after childbirth at the second week postnatal follow-up.

All participants involved in the study provided their informed consent before completing the surveys, and they came from all over China. Data were collected from 1 December 2021 to 1 July 2022. According to the statistics department of the hospital, there are approximately 8 to10 new mothers in a maternity ward per day. The study sample consisted of mothers who were: (1) over 18 years old, (2) delivered a single-born full-term baby with a neonatal Apgar score ≥ 8, (3) had no breast diseases, (4) were able to stay in the same room with the baby after birth, (5) were able to understand the study and the instruments involved, (8) had a short hospital stay of ≤ 4 days, and (9) were informed about the purpose of the study and its circumstances in advance and provided informed consent to participate. The exclusion criteria were as follows, mothers who had cognitive impairment or mental illness who could not establish meaningful communication, or mothers who were prohibited from breastfeeding due to medical factors or diseases such as taking drugs that affect breastmilk within two weeks after delivery. The sample size was determined based on the rule that the sample should contain 5 to10 mothers for each item. Assuming a 10% rate of invalid questionnaires, 237 mothers were recruited in this study, and 206 mothers completed the questionnaire for data collection. Mothers excluded from the study included those who rejected breastfeeding and could not be reached because they gave wrong or unused phone numbers or refused to fill out the questionnaires again.

**Fig. 1:**
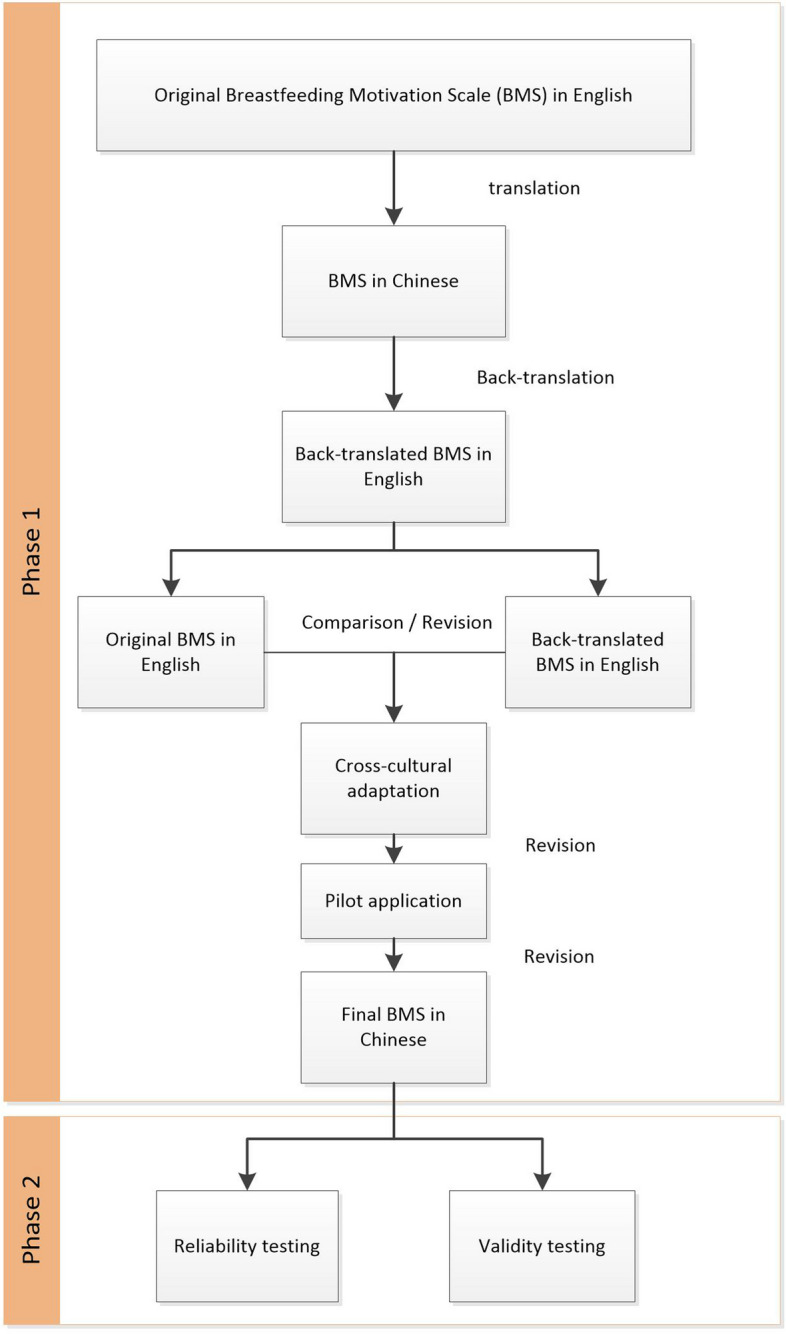
Flow chart of the study procedures

To translate the BMS into Chinese and assess its psychometric properties among Chinese mothers during the postpartum period, the study was conducted in two phases: (1) comprehensive translation, back-translation, cross-cultural adaptation, and pretest; and (2) reliability testing, such as testing of the internal consistency and test–retest reliability following translation and the content validity, construct validity, and convergent and discriminant validity. The flow chart of the study procedures is shown in Fig. 1.

### Ethical considerations

Written permission was obtained via email from Ariel University (Israel) Lecturer Miri Kestler–Peleg, the author who developed the original version of the BMS. The study was approved by the Human Research Ethics Committee of Shanghai First Maternity and Infant Hospital in Shanghai, China (No. KS22340 from 1 December 2021). The study complied with the ethical principles of the World Medical Association Declaration of Helsinki principles.

### Procedures

#### Phase 1-Translation, back-translation, cross-cultural adaptation and pilot application

The final English version of the BMS was clarified with the author via email. The translation of the BMS closely followed the guidelines of the principles of good practices for translation and cultural adaptation of measures established by the International Society for Pharmacoeconomics and Outcomes Research (ISPOR) [26]. Phase 1 involved four steps. Step (1) Translation: Two bilingual native Chinese speakers who were also fluent in the English language were asked to independently translate the English version of the BMS and the accompanying instructions provided with the scale from the English to Chinese language. The two translators were a Ph.D. in nursing who worked in the US and a Ph.D. in obstetrics who had returned from studying in the US. The two translations were then assessed by two native Chinese speakers online, who reached a consensus on any discrepancies to produce a single translated scale in Chinese named Chinese version 1 of the BMS. Step (2) Back-translation: Two translators with knowledge of both the Chinese language and culture independently back-translated the Chinese version 1 of the BMS to English. The two translators were a Ph.D. in obstetrics and gynecology nursing who studied and worked in the United States and an English teacher from a popular university in the U.S. The two translators discussed the discrepancies of the back-translation and reached a consensus. Then, they synthesized the details of the back-translation to produce a single back-translated scale in English, which was named the back-translation version 1 of the BMS. The researchers sent the Chinese version 1 of the BMS and the back-translated version 1 of the BMS to the original author of the BMS to ask for his advice. The original author proposed changes in three items of the scale, and two modifications (“among us” represents the closest circle in item 3 and only mothers can breastfeed the baby in item 10) were made based on the author’s advice. Then, we reached a consensus to produce Chinese version 2 of the BMS. Step (3) For cross-cultural adaptation, five experts who majored in obstetrics and were skilled at translation (a nursing specialist familiar with cross-cultural adaptation, a nursing management specialist, an obstetric nursing specialist, an obstetrical clinician and an international board-certified lactation consultant) were invited to evaluate the accuracy of the translation, whether the translated version was clear and easy to understand, and whether it was consistent with the cultural background of Chinese people. The experts rated each item on a 4-point Likert scale ("very relevant" = 4, "relevant" = 3, "not very relevant" = 2, "not relevant at all" = 1). They provided few comments, and no further modifications were needed. Then, Chinese version 3 of the BMS was produced. Step (4) To perform an initial evaluation and to assess the understanding of the Chinese version 3 of the BMS in the Chinese population, a small sample pilot study was conducted. Twenty-five native-Chinese-speaking mothers were recruited after labor at the hospital according to the inclusion and exclusion criteria to fill out a sample scale (Chinese version 3 of the BMS). Cognitive interviews were also conducted to collect modification suggestions from respondents. Cognitive interviews are a common method used to pretest whether survey questions are understandable and answerable. The 25 mothers were encouraged to describe their thoughts while answering the scale questions, which could be done through think aloud, verbal probing, and paraphrasing methods, etc. [27]. We developed a semi structured interview guide to collect further information about the scale from the women. The interview questions were as follows: Can you repeat this question in your own words? What does that word mean to you? Tell me more about your thinking when you think about the question. Are there questions you believe should be modified? Why? What do you think the word in ‘...’ could be adjusted to make the question more understandable? Are there questions you believe should be deleted? Why? Any other questions? The interviews were recorded via audio, were held in the meeting room of the ward and were conducted by an investigator who was trained in cognitive interviewing. Then, the translation team modified Chinese version 3 of the BMS according to the interview content, which the investigator summarized after the interviews. Generally, respondents reported that the sample scale could not be misunderstood, and four slight changes of items were made after the interviews (see Table 1 for the modifications made after the interviews). Finally, Chinese version 4 of the BMS was formed. This was the final Chinese version of the BMS before psychometric property testing. We call Chinese version 4 of BMS ‘the Chinese version of the BMS’ below.

**Table 1:** Modifications made to create the Chinese version of the BMS after cognitive interviews

Original BMS in English	Chinese version 3 of the BMS	Chinese version 4 of the BMS	Rationale
It's been said that breastfeeding is good for the baby's immune system	据说母乳喂养有益于宝宝的免疫系统。	我听说母乳喂养对宝宝的免疫系统有好处。	It was difficult for three respondents to understand that this is a self-administered scale. Therefore, it was changed from "据说" to "我听说". "有益于" and "有好处" had the same meaning, but the latter was more preferred and was more consistent with the style of the Chinese scale
It makes me feel special that this role is exclusively mine	母乳喂养这个能力只属于我让我觉得很特别。	母乳喂养让我感到我是不可替代的, 因为只有我能做到。	Three respondents wondered about the word "特别". Analysis of the original item showed that "很特别" meant "不可替代". Therefore, we directly used the hidden meaning
This way I strengthen my psychological and physical attachment to my baby	母乳喂养增强了我与宝宝心理和生理间的依恋关系。	母乳喂养拉近了我和我宝宝生理和心理的距离。	Four respondents believed that babies were not naturally attached to their mothers strongly. Therefore, "依恋" was replaced by "距离" to avoid subjective opinions on objective situations
It seems natural to me to breastfeed a baby who was nurtured in my body before it was born	对我来说, 母乳喂养一个在我体内孕育出来的孩子是件很自然的事。	我觉得母乳喂养自己生的宝宝是一件天经地义的事。	Four respondents questioned "自然", especially in the whole item. Therefore, it was replaced by "天经地义" (a Chinese idiom); this expression is more consistent with a traditional Chinese understanding of breastfeeding

#### Phase 2 – Testing of the psychometric properties of the Chinese version of the BMS

After obtaining permission to carry out the study from the human research ethics committee of Shanghai First Maternity and Infant Hospital, we oriented the investigators and registered nurses working in Shanghai First Maternity and Infant Hospital to clarify the details of the survey, including the team members, survey tools, time, communication method, data collection methods, and so on. The questionnaires were administered to the mothers from 1 December 2021 to 1 July 2022. To avoid affecting the clinical work of the hospital and the nonparticipating mothers, we collected the questionnaires from 3:00 to 5:00 p.m. to reduce the possibility of survey interruption and to reduce the number of invalid questionnaires. Before the survey began, the investigators explained the study protocol to the mothers again. The mothers were instructed on how to understand the questionnaires and how to complete them.

The questionnaires were collected and checked by the investigators on site, i.e., in the maternity wards. The mothers completed an online version of the self-administered questionnaires. The mothers were asked to scan a QR code to fill out the questionnaire, and all responses were anonymous. It took approximately 15 min to complete the questionnaire, and this amount of time was assessed as adequate. Twenty mothers were randomly selected to answer the Chinese version of the BMS twice, with second time being 2 weeks after the first to assess the test–retest reliability during the postpartum 2-week visit. This 2-week duration was proposed by Stewart and Bass because it is difficult to affect a person's memory and practice in 2 weeks [28]. The participating mothers’ telephone numbers and gestational age were recorded when the data were collected for the first time. We invited the five experts for the two rounds of expert consultation to test the content validity as mentioned above. All experts had bachelor's degrees or above.

### Statistical analysis

Statistical analyses were performed using Excel, SPSS 24.0, and Amos 22.0. Reliability was assessed in two ways: test–retest reliability and internal consistency. It was important to ensure that the assessment reflected the mothers’ true breastfeeding motivation. Test–retest reliability was assessed by item-by-item testing. The instrument was reapplied in approximately 10% of the sample 2 weeks later. The intraclass correlation coefficient (ICC) was calculated. The closer the ICC of the scale is to 1, the higher the stability of the scale is, indicating that the instrument is more reliable. The ICC was ≥ 0.75, indicating that the test–retest reliability of the scale was good. The internal consistency of the scale was analyzed with Cronbach’s α. Values ≥ 0.70 are considered acceptable [29]. Content validity was tested using a content validity index, including scale-level CVI (S-CVI) and item-level CVI (I-CVI). These items were revised and supplemented through two rounds of expert consultation above to verify the content validity. Confirmatory factor analysis (CFA) was employed to analyze the construct validity, convergent validity and discriminant validity. The suitability of the dataset was verified through the Kaiser‒Meyer‒Olkin measure (KMO) (> 0.50) and Bartlett’s test of sphericity (*P* < 0.05). The parameters were carried out using the maximum likelihood estimation method [30]. The goodness of fit of the model was evaluated using the following statistics and the minimum standards of indices: (a) the standardized χ2(CMIN/*df*) (chi-square mean/degree of freedom) should be lower than 3.0 [31], (b) the root mean square error of the approximation (RMSEA) should be lower than 0.1 [32], and (c) the comparative fit index (CFI), incremental fit index (IFI), and Tucker-Lewis index (TLI) should all be ≥ 0.90 [31, 33]. Convergent validity refers to the similarity of measurement results when using different measurement methods to measure the same goal. Convergent validity was evaluated by the average variance extracted (AVE) and composite reliability (CR). Discriminant validity means that the observed values should be distinguishable from each other when measuring different indicators. The discrimination validity is generally tested by comparing the AVE square root with the phase relation value. The level of significance was set at < 0.05.

## Results

### Sociodemographic characteristics

The demographic characteristics of the mothers are shown in Table 2. A total of 206 mothers were involved in this study; the oldest mother was 44 years old, and the youngest mother was 23 years old. The mean age was 31.05 ± 3.89.

**Table 2:** Demographic characteristics of the mothers (*n* = 206)

Variables	*N* (%)
**Gravidity**	
1	84 (40.8)
2	70 (34.0)
3 or more	52 (25.3)
**Parity**	
Primiparity	144 (69.9)
Multiparity	62 (30.10)
**Mode of birth**	
Vaginal delivery	91 (44.2)
Cesarean Section	115 (55.8)
**Infant's Apgar score within 1 min**	
8	7 (3.4)
≥ 9	199 (96.6)
**Pregnancy complications**	
None	174 (84.5)
One complication or more	32 (15.5)
**Variables**	$\overline{{\varvec{X}}}\pm {\varvec{S} }$
**Gestational age**	38.19 ± 1.19 weeks
**Infant birthweight**	3287.52 ± 431.18 g

The number of participants of Han nationality was 199 (96.6%), while 7 participants were not of Han nationality (3.4%) (e.g., Tibetan, Zhuang, and Yue).

Nonreligious persons accounted for 97.6%, and religious persons accounted for 2.4% (e.g., Christian, Catholic, and Buddhist).

A total of 95.1% of participants' caregivers during the hospital stay were their husbands, 3.4% of them were their mothers, and 1.5% of them were their mothers-in-law.

A total of 99.5% of the participants were married, and 0.5% of the participants were remarried.

A total of 5.3% of the participants had a junior high school degree or below; 14.6% of those had a senior high school/technical secondary school education, 60.2% of those had a bachelor’s degree or had an associate’s degree, and 19.9% of those had a master's degree or above.

The distribution of the family’s monthly income showed that 5.3% of the participants had a monthly family income ≤ 5000 RMB, 15.0% of the participants had an income of 5001–10000 RMB per month, 26.2% of them had an income of 10,001–15000 RMB per month, and 53.4% of them had an income ≥ 15001 RMB a month. The distribution of occupations showed that 95.7% of mothers were employed and that 4.3% of the participants were unemployed.

### Reliability and validity

#### Internal consistency and test–retest reliability

The internal consistency coefficient [28] of the Chinese version of the BMS measured by Cronbach's α was 0.887. The Cronbach's α values of the subscales of the Chinese version of the BMS were 0.876, 0.821, 0.857, 0.900, and 0.849, respectively. These values revealed that the Chinese version of the BMS was acceptable for all subscales.

The scale was administered twice to 20 mothers with an interval of two weeks to evaluate time durability. The ICC for 20 mothers with a two-week interval was 0.897 (*P* < 0.001), indicating adequate stability over time.

#### Content validity and construct validity

The validity of the Chinese version of the BMS was tested using expert consultation and confirmatory factor analysis.

After two rounds of expert consultation, the content validity of the scale was calculated. The item-level content validity index (I-CVI) of each item in the Chinese version of the BMS ranged from 0.8 to 1.0, while the scale-level content validity index (S-CVI) was 0.83, with a scale-level content validity index average (S-CVI/Ave) of 0.97.

The hypothesized measurement model of the Chinese version of the BMS comprised 23 items across 5 factors: enjoyment and bonding (8 items), maternal self-perception (6 items), significant others’ pressure (4 items), baby’s health (2 items) and instrumental needs (3 items). The Kaiser‒Meyer‒Olkin value was 0.952, with *P* < 0.001, and the significance of Bartlett's sphericity was 0.000 (χ^2^ = 3680, *df* = 253). These results supported proceeding with the factor analysis.

**Fig. 2:**
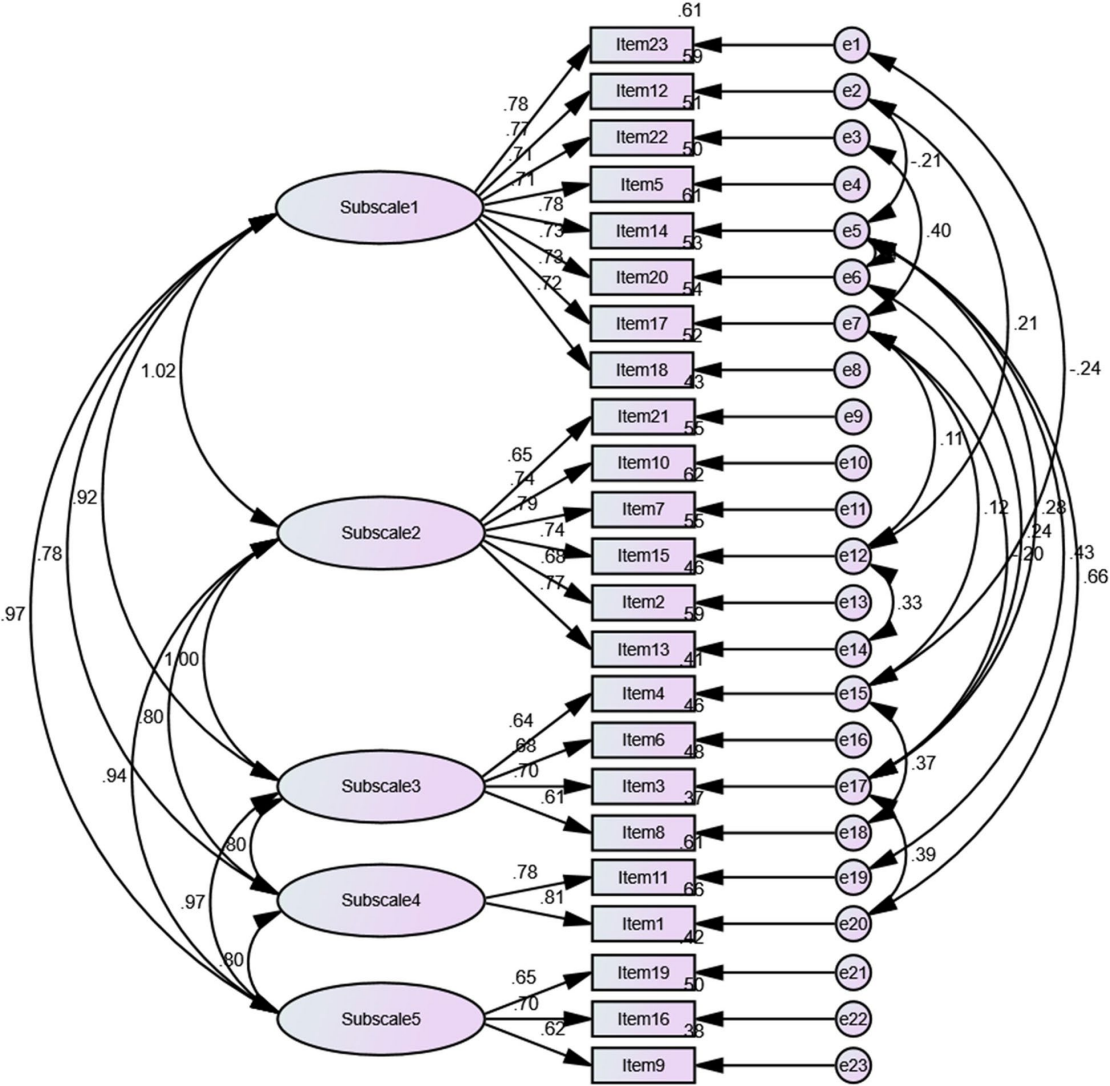
Confirmatory factor analysis of the Chinese version of the BMS. Note. subscale1 = Enjoyment and bonding, subscale 2 = Maternal self-perception, subscale 3 = Significant others’ pressure, subscale 4 = Baby’s health, subscale 5 = Instrumental needs

The construct validity, convergent validity and discriminant validity were estimated using the maximum likelihood estimation method [30]. A 5-dimensional model identical to the structure of the original version of the BMS was proposed with the aim of checking whether the model was adequate. The goodness of fit statistics was χ^2^/*df* = 2.4, *P* < 0.001, RMSEA = 0.08, CFI = 0.91, IFI = 0.92, and TLI = 0.90, indicating an acceptable model fit. The standardized factor loadings were all greater than 0.60 (*P* ≤ 0.001) (Fig. 2). All the items loaded significantly onto their respective factors. No variables were deleted from the original model in this study.

As a type of construct validity, convergent validity is related to whether a latent variable is well estimated with the selected indicators (i.e., tested variables should have high correlations with supposed similar constructs) [34]. Statistical evidence of convergent validity was checked through the convergent validity coefficient (i.e., composite reliability) and average variance extracted (AVE) [35, 36]. The formula $\frac{{(\sum Factor\;loading\;value)}^2}{{(\sum\;Factor\;loading\;value)}^2+\sum\;measurement\;Error}$ was used to calculate the composite reliability (CR), and the formula $\frac{{\sum\;Factor\;loading\;value}^2}{{\sum\;Factor\;loading\;value}^2\;+\sum\;measurement\;Error}$ was used to calculate the AVE [34].

The CR has to be 0.70 or greater and the AVE has to be 0.36 or greater for acceptable convergent validity [37, 38]. As shown in Table 3, the CR values corresponding to each latent variable were 0.733 ~ 0.926, and the AVE values were 0.476 ~ 0.653.

**Table 3:** Convergent validity of the Chinese version of the BMS

Paths			Estimate	AVE	CR
Item 23	< –	Enjoyment and bonding	0.793	0.581	0.926
Item 12	< –	Enjoyment and bonding	0.803		
Item 22	< –	Enjoyment and bonding	0.765		
Item 5	< –	Enjoyment and bonding	0.745		
Item 14	< –	Enjoyment and bonding	0.784		
Item 20	< –	Enjoyment and bonding	0.740		
Item 17	< –	Enjoyment and bonding	0.795		
Item 18	< –	Enjoyment and bonding	0.738		
Item 21	< –	Enjoyment and bonding	0.690		
Item 10	< –	Maternal self-perception	0.782	0.608	0.885
Item 7	< –	Maternal self-perception	0.818		
Item 15	< –	Maternal self-perception	0.798		
Item 2	< –	Maternal self-perception	0.694		
Item 13	< –	Maternal self-perception	0.799		
Item 4	< –	Significant others’ pressure	0.687	0.476	0.784
Item 6	< –	Significant others’ pressure	0.710		
Item 3	< –	Significant others’ pressure	0.720		
Item 8	< –	Significant others’ pressure	0.640		
Item 11	< –	Baby’s health	0.801	0.653	0.790
Item1	< –	Baby’s health	0.815		
Item 19	< –	Instrumental needs	0.670	0.479	0.733
Item 16	< –	Instrumental needs	0.750		
Item 9	< –	Instrumental needs	0.653		

Discriminant validity is also a type of construct validity. It demonstrates that different or unique constructs do not correlate with each other or have low correlations with tests from which it should differ [36, 39]. The discriminant validity was used to determine whether the five indicators of ‘enjoyment and bonding’, ‘maternal self-perception’, ‘significant others’ pressure’, ‘baby’s health’ and ‘instrumental needs’ were distinct factors from one another for the current study. Generally, discriminant validity was assessed through confirmatory factor analytic models with every pair of latent constructs using AMOS [40]. To verify the discriminant validity of the Chinese version of the BMS, the correlations among the five measured variables needed to be lower than Pearson *r* = 0.85 [34], and the square root of average variance extracted (AVE) from the variable needed to be greater than the interconstruct correlations between the variable and other variables in the model [35]. Table 4 provides strong evidence of discriminant validity, with correlations among variables (*P* < 0.001) (off-diagonal elements) and the square root of AVE on the diagonal.

**Table 4:** Discriminant validity of the Chinese version of the BMS

	**Enjoyment and bonding**	**Maternal self-perception**	**Significant others’ pressure**	**Baby’s health**	**Instrumental needs**
Enjoyment and bonding	***0.762***				
Maternal self-perception	0.053*	***0.779***			
Significant others’ pressure	0.050*	0.055*	***0.690***		
Baby’s health	0.042*	0.044*	0.044*	***0.808***	
Instrumental needs	0.047*	0.046*	0.048*	0.039*	***0.692***

^*^Indicates *P* < 0.001; italicized bold diagonal elements are the square root of average variance extracted (AVE)

## Discussion

This is the first study to adapt the BMS into a Mandarin version among Chinese mothers during the postnatal period in mainland China. In the current study, the translation and verification of the psychometric properties of the Chinese version of the BMS were conducted according to a standardized procedure. After the mixed-method approach, a cognitive interview was performed to finalize the Chinese version of the scale before testing the psychometric properties. A few modifications of the items were made apart from clarifying some meanings of Chinese words. The positive feedback from the pilot application suggested that the Chinese version of the BMS was adapted to the Chinese context and achieved conceptual equivalence after the developer's final approval.

Reliability refers to the degree of consistency of the results of a scale across different times, investigators, populations and scenarios [41], and it is mainly evaluated by internal consistency and test–retest reliability. The results of this study revealed that the Cronbach’s α was 0.887 for the overall scale, which was similar to the values found for the original version [20] (values of 0.62—0.93) and in the Turkish version (values of 0.658—0.879) [23]. A Cronbach’s α of 0.80—0.90 indicates that a scale’s internal consistency is outstanding [41]. The ICC of the overall scale demonstrated that the Chinese version of the BMS has good test–retest reliability. Overall, the reliability analysis results indicate that the Chinese version of the BMS is free from measurement error over time.

Our research team adopted the Chinese BMS to conduct an on-site survey of Chinese mothers to analyze its validity, including its content validity, convergent validity, discriminant validity and construct validity. Content validity is considered an important measurement property referring to the accuracy of the item content to achieve the expected measurement results (I-CVI ≥ 0.78, S-CVI ≥ 0.8, and S-CVI/Ave ≥ 0.9) [41]. In our study, the I-CVI was 0.80 ~ 1.00, the S-CVI was 0.83, and the S-CVI/Ave was 0.97, indicating that the content validity of Chinese BMS was good. The CFA of this study showed an adequate fit for the structure of 5 factors, consistent with the original version of the BMS [20]. We carried out a CFA to determine whether the scores reproduced the structure of the 5 dimensions of the Israeli version of the BMS [42]. The results of our study showed that the model fit successfully. Construct validity reflects the degree of integration between the questionnaire structure and the framework on which it is based, which requires item loadings that are over 40 [28]. The results of the CFA, including representative indices reflecting construct validity, convergent validity and discriminant validity, indicated a good fit to a multidimensional model, and there was good correspondence between the factors and the measurement items. In addition, convergent validity was demonstrated by associations between 5 factors (*P* < 0.001), and good discriminability was found for all constructs.

Chinese mothers tend to live with their in-laws after giving birth, and breastfeeding is often influenced by the older generation. This may be different in other countries. In addition, most Chinese women, especially those living and working in Shanghai, have a high education level and work pressure. They have received a range of information about feeding their babies from the Internet, books and other media, and breastfeeding is easily affected by various aspects. Additionally, there are some obstacles, such as a lack of supportive environment and professional support [43]. A question that needs to be further explored is whether the different dimensions of motivation explored by the breastfeeding motivation scale constructed solely based on SDT are comprehensive? We can hypothesize whether it is possible to construct a more comprehensive breastfeeding motivation scale based on multiple motivation theories to explore more breastfeeding motivations.

Overall, there is sufficient reliability and validity evidence to support the use of the Chinese version of the BMS in Chinese mothers. We believe that the Chinese BMS will offer an opportunity to explore Chinese mothers’ motivation for breastfeeding in the Chinese context and to make a significant contribution to improving exclusive breastfeeding rates by promoting Chinese mothers’ breastfeeding motivation.

However, there are some limitations; for example, this study recruited women from only one hospital, and breastfeeding motivation was investigated at only two time points. In the future, we will consider expanding the sample size to recruit pregnant women and conduct research at multiple time points and multiple research institutions after delivery. Based on motivation theory, we will collaborate with the research team of the original BMS (we have contacted the original developer and reached an agreement) to compare the characteristics of postpartum mothers in China and Israel, update or improve the BMS, and test its validity and reliability.

Our findings showed that the Chinese version of the BMS is a reliable and valid tool for evaluating the breastfeeding motivation of mothers during lactation.

## Conclusions

This is the first study to translate the BMS into Mandarin Chinese and assess psychometric properties of the Chinese version of the BMS. This study has shown that the Chinese version of the BMS has acceptable psychometric characteristics and that it is suitable for measuring breastfeeding motivation of hospitalized mothers during their postpartum period in mainland China. Use of the Chinese version of the BMS should promote and facilitate greater collection of standardized data on breastfeeding motivation of mothers in China. In the future, we will compare breastfeeding motivation at different time points in the perinatal period to explore the period of weak breastfeeding motivation, and then carry out targeted interventions for mothers.

### Supplementary Information

**Additional file 1.** \English BMS.docx.

**Additional file 2.** \Chinese BMS.docx.

## Data Availability

The datasets used and analyzed during the current study are available from the corresponding author upon reasonable request.

## Abbreviations

BMS: Breastfeeding Motivation Scale
ISPOR: International Society for Pharmacoeconomics and Outcomes Research
ICC: Intraclass correlation coefficient
I-CVI: Item-level content validity index
CFA: Confirmatory factor analysis
CFI: Comparative fit index
IFI: Incremental fit index
TLI: Tucker-Lewis index
AVE: Average variance extracted
CR: Composite reliability
