# Supplementary material for: Translation and validation of the Breastfeeding Motivation Scale in China
Source: Int Breastfeed J. 2024 Jan 4;19:2. doi: 10.1186/s13006-023-00610-z (PMC10768438; doi:10.1186/s13006-023-00610-z)
Supplement: Supplementary file 1 — Additional file 1. \English BMS.docx. [file 13006_2023_610_MOESM1_ESM.docx]

**Breastfeeding Motivation Scale**

Women breastfeed their babies for various reasons. We would like to know the reasons you decided to breastfeed your baby. The following items describe different reasons for breastfeeding “. To what extent do you agree with the reasons specified? I breast-feeding because… . (1 ¼ Strongly Disagree; 4 ¼ Strongly Agree).

1. It's been said that breastfeeding is good for the baby's immune system.
2. It feels good that my baby depends on me.
3. Among us, this is what is acceptable.
4. By breastfeeding, people who are close to me appreciate me more.
5. It's fun to breastfeed.
6. I would be ashamed if I did not breastfeed.
7. I feel proud and important while breastfeeding.
8. This way people who are close to me won't criticize or bother me.
9. Breastfeeding saves money.
10. It makes me feel special that this role is exclusively mine.
11. It is healthy for my baby.
12. I feel good while breastfeeding.
13. My life has a purpose while I'm breastfeeding.
14. This way I strengthen my psychological and physical attachment to my baby.
15. I like myself better if I breastfeed.
16. It's good for my health-it prevents osteoporosis, reduces bleeding, and prevents cancer.
17. Breastfeeding gives me a sublime sensation.
18. It seems natural to me to breastfeed a baby who was nurtured in my body before it was born.
19. This way I can lose weight faster.
20. I want to be close to my baby.
21. I like to think that I'm giving him/her a part of myself.
22. It excites me to breastfeed.
23. Breastfeeding makes me happy.
