# Supplementary material for: Translation and validation of the Breastfeeding Motivation Scale in China
Source: Int Breastfeed J. 2024 Jan 4;19:2. doi: 10.1186/s13006-023-00610-z (PMC10768438; doi:10.1186/s13006-023-00610-z)
Supplement: Supplementary file 2 — Additional file 2. \Chinese BMS.docx. [file 13006_2023_610_MOESM2_ESM.docx]

**知情同意书**

尊敬的朋友：

您好！我是上海市第一妇婴保健院东院产科4B病区的护士余燕飞，现正进行“基于自我决定理论的早期母乳喂养中断的影响因素研究”。

本研究为横断面调查，调查的目的是您产后母乳喂养进行情况，并探究您母乳喂养情况影响因素，为后续为您提供进一步的医疗护理帮助打基础。

参与本研究不会损害您的任何权益，衷心希望您可以参与本研究。若您同意，我们将发一份问卷给您，希望您客观如实填写并完成。填写过程中若您对问卷仍存有疑问，可以向发送问卷的研究人员咨询。另外，您需要在问卷相应位置**写下您的手机号码**，我们**14天后还会致电您再次进行同样的调查，**因此，麻烦您**存一下我们病区的电话** ，方便联系。

您的个人资料我们将会保证其安全保密，且您的姓名在研究过程中或研究报告公开发表时均不会被提及。

您可以选择不参加本研究，或在任何时候通知研究者要求退出研究，您的个人数据将不被纳入研究结果，您的任何医疗待遇与权益也不会因此而受到影响。

若您想了解与本研究有关的信息资料和研究进展，或您有关于本项研究参加者权益方面的问题，您都可以通过 与 余燕飞 联系。再次感谢您的参与。

祝身体健康，家庭幸福！

一妇婴东院产科4B病区的护士 余燕飞

2021年 11 月 17 日

知情同意书

本人已经详细阅读完这份知情同意书，本人已获知这项研究对本人及本人孩子的身体没有伤害、对本人及本人孩子的治疗和护理没有影响。本人自愿同意参加这项研究。

签名：____________

日期：____________

**一、一般情况调查表**

联系方式：

编号：

**一、产妇情况**

1. 年龄_______岁
2. 民族：①汉族 ②其他_______
3. 宗教：①无 ②其他_______
4. 与陪护者关系： ①夫妻 ②母女 ③婆媳 ④其他_______
5. 婚姻状况：①在婚 ②再婚 ③离异 ④其他_______
6. 文化程度：①初中及以下 ②高中、中专 ③本科、大专④硕士及以上
7. 家庭月收入（元/月）：①≤5000 ②5001-10000 ③10001-15000 ④≥15001
8. 职业： _______
9. 孕产次：G P **（医务人员填）**
10. 孕周：_______周**（医务人员填）**
11. 分娩方式**（医务人员填）**：①顺产 ②剖宫产 ③阴道助产
12. 疾病情况**（医务人员填）**：①健康 ②GDM ③妊娠期高血压疾病 ④妊娠合并贫血 ⑤妊娠合并肝功能受损 ⑥其他_______

**二、新生儿情况（医务人员填）**

1. 出生体重：_______ 克
2. Apgar评分：_______ 分

**二、 母乳喂养动机量表**

**指导语**：母乳喂养孩子可能有各种各样的原因。我们想知道您决定母乳喂养您孩子的原因。以下条目描述了母乳喂养的不同理由。您在多大程度上同意下面的理由?根据您自己的想法在下面不同程度选项上“√”进行选择。

| 1、我听说母乳喂养对宝宝的免疫系统有好处。 | 非常不同意 | 不太同意 | 同意 | 非常同意 |
| --- | --- | --- | --- | --- |
| 2、我的宝宝依赖我让我感觉很好。 | 非常不同意 | 不太同意 | 同意 | 非常同意 |
| 3、我们中的大多数人是可以接受母乳喂养的。 | 非常不同意 | 不太同意 | 同意 | 非常同意 |
| 4、因为母乳喂养宝宝，身边的亲朋好友更加赞赏我。 | 非常不同意 | 不太同意 | 同意 | 非常同意 |
| 5、母乳喂养很有乐趣。 | 非常不同意 | 不太同意 | 同意 | 非常同意 |
| 6、如果我不母乳喂养的话，我会感到很惭愧。 | 非常不同意 | 不太同意 | 同意 | 非常同意 |
| 7、母乳喂养让我感到自豪和重要。 | 非常不同意 | 不太同意 | 同意 | 非常同意 |
| 8、如果我母乳喂养的话，身边的亲朋好友就不会批评我、烦扰我。 | 非常不同意 | 不太同意 | 同意 | 非常同意 |
| 9、母乳喂养省钱。 | 非常不同意 | 不太同意 | 同意 | 非常同意 |
| 10、母乳喂养让我感到我是不可替代的，因为只有我能为我的宝宝做到。 | 非常不同意 | 不太同意 | 同意 | 非常同意 |
| 11、母乳喂养对宝宝来说是健康的。 | 非常不同意 | 不太同意 | 同意 | 非常同意 |
| 12、母乳喂养时我感觉很好。 | 非常不同意 | 不太同意 | 同意 | 非常同意 |
| 13、母乳喂养时我感觉我的生活有了目标。 | 非常不同意 | 不太同意 | 同意 | 非常同意 |
| 14、母乳喂养拉近了我和我宝宝生理和心理的距离。 | 非常不同意 | 不太同意 | 同意 | 非常同意 |
| 15、我更喜欢母乳喂养时的自己。 | 非常不同意 | 不太同意 | 同意 | 非常同意 |
| 16、 母乳喂养对我的健康有好处，可以预防骨质疏松，减少产后出血，预防癌症。 | 非常不同意 | 不太同意 | 同意 | 非常同意 |
| 17、母乳喂养给我一种崇高的感觉。 | 非常不同意 | 不太同意 | 同意 | 非常同意 |
| 18、我觉得母乳喂养自己生的宝宝是一件天经地义的事。 | 非常不同意 | 不太同意 | 同意 | 非常同意 |
| 19、母乳喂养可以帮我更快减肥。 | 非常不同意 | 不太同意 | 同意 | 非常同意 |
| 20、我想亲近我的宝贝。 | 非常不同意 | 不太同意 | 同意 | 非常同意 |
| 21、我认为母乳喂养意味着我把我的一部分给了宝宝。 | 非常不同意 | 不太同意 | 同意 | 非常同意 |
| 22、母乳喂养让我兴奋。 | 非常不同意 | 不太同意 | 同意 | 非常同意 |
| 23、母乳喂养让我感到幸福。 | 非常不同意 | 不太同意 | 同意 | 非常同意 |
